# Mastiha (*Pistacia lentiscus*) Improves Gut Microbiota Diversity, Hepatic Steatosis, and Disease Activity in a Biopsy‐Confirmed Mouse Model of Advanced Non‐Alcoholic Steatohepatitis and Fibrosis

**DOI:** 10.1002/mnfr.201900927

**Published:** 2019-10-23

**Authors:** Aimo Kannt, Efstathia Papada, Claire Kammermeier, Giuseppe D'Auria, Nuria Jiménez‐Hernández, Martin Stephan, Uwe Schwahn, Andreas Nygaard Madsen, Mette Viberg Østergaard, George Dedoussis, M. Pilar Francino, for the MAST4HEALTH consortium

**Affiliations:** ^1^ Sanofi Research and Development 65926 Frankfurt Germany; ^2^ Institute of Experimental Pharmacology, Medical Faculty Mannheim University of Heidelberg 68167 Mannheim Germany; ^3^ Harokopio University Athens Kallithea 17676 Greece; ^4^ Foundation for the Promotion of Health and Biomedical Research (FISABIO) 46035 Valencia Spain; ^5^ Gubra 2970 Horsholm Denmark

**Keywords:** Chios mastic gum, fibrosis, gut microbiome, Mastiha, NASH, non‐alcoholic steatohepatitis

## Abstract

**Scope:**

As a result of the obesity epidemic, the prevalence of non‐alcoholic steatohepatitis (NASH) is increasing. No drug is approved for the treatment of NASH. In this study, the effect of a nutritional supplement, Mastiha or Chios mastic gum, on metabolic and histological parameters and on the gut microbiome in mice with NASH and fibrosis was investigated.

**Methods and results:**

Advanced NASH was induced by feeding C57BL/6J mice a diet rich in fat, sucrose, and cholesterol for 41 weeks. After randomization, animals received the NASH‐inducing diet with or without 0.2% (w/w) Mastiha for a further 8 weeks. Disease activity was assessed by liver histology and determination of plasma transaminase activities. Fecal microbiota DNA extraction and 16S rRNA amplicon sequencing were used to determine the composition of the gut microbiome.

Mastiha supplementation led to a significant reduction in circulating alanine aminotransferase (ALT) activity, improvement in hepatic steatosis and collagen content, and a reduction in NAFLD activity score. Furthermore, it resulted in a partial but significant recovery of gut microbiota diversity and changes in identity and abundance of specific taxa.

**Conclusion:**

This is the first study demonstrating an improvement in disease activity in mice with advanced NASH with fibrosis by a diet containing Mastiha.

## Graphical abstract

As a result of the obesity epidemic, the prevalence of non‐alcoholic fatty liver disease (NAFLD) is increasing. No drug is approved for the treatment of NAFLD. It has been demonstrated that a nutritional supplement, Chios Mastic Gum, can improve liver function and partially normalize gut microbiota composition in mice with advanced NAFLD, hepatic inflammation, and liver fibrosis.

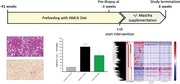

## 1 Introduction

Nonalcoholic fatty liver disease (NAFLD) represents a wide spectrum of liver abnormalities including hepatic steatosis, non‐alcoholic steatohepatitis (NASH) characterized by lobular inflammation and hepatocyte ballooning, and liver fibrosis. NASH with fibrosis is a strong risk factor for the development of cirrhosis or hepatocellular carcinoma, and advanced hepatic fibrosis is linked to an increase in overall and liver‐related mortality.^1^ NAFLD is very common, hepatic steatosis is estimated to be present in >25% of the global population.^2^ There is a strong association of NAFLD with other cardio‐metabolic conditions such as obesity, type‐2 diabetes, hypertension, and dyslipidemia.^3^, ^4^ There is currently no pharmacotherapy approved for the treatment of advanced stages of NAFLD. First‐line treatment consists of diet and exercise. However, some experimental drugs are currently in advanced stages of clinical development.^5^ Sustained weight loss, via lifestyle modification,^6^ bariatric surgery,^7^ or pharmacological intervention with a glucagon‐like peptide 1 (GLP1)‐receptor agonist^8^ has been demonstrated to lead to NASH resolution and prevention of fibrosis progression in obese individuals with NASH.

NAFLD is a multifactorial, heterogeneous, slowly chronically progressing disease. Its pathophysiological mechanisms are incompletely understood and differ between individuals. Likely, genetic predisposition and multiple “hits” such as insulin resistance, adipose tissue hormones, dietary factors, gut microbiota, or epigenetic factors determine the likelihood of progression from simple hepatic steatosis, a comparably benign condition, to advanced stages of NASH and fibrosis.^9^

Thus, pleiotropic mechanisms simultaneously targeting several pathways playing a role in NASH development may have a higher probability of preventing the progression of this multifactorial disease. For example, natural food supplements such as vitamin E, omega‐3 fatty acids, phenolic compounds, and other phytochemicals with a broad spectrum of activities are currently under investigation as potential non‐pharmacological approaches in NAFLD.^10^, ^11^

Mastiha or Chios mastic gum is a natural food supplement, which is obtained as a resin from the stems and the branches of the shrub *Pistacia lentiscus*, exclusively cultivated in Chios Island, Greece. According to the European Medicines Agency, Mastiha has been recognized as a traditional herbal medicinal product used in mild dyspeptic disorders, in symptomatic treatment of minor skin inflammations and in healing of minor wounds.^12^ Due to its high content in terpenic acids, mainly triterpenes such as mastihadienonic, isomastihadienonic, olealonic, and moronic acids,^13^, ^14^ Mastiha exhibits anti‐inflammatory,^15^ antioxidant,^16^ cytotoxic,^17^ lipid‐lowering properties,^18^ as well as beneficial effects on the gastrointestinal system.^19^, ^20^ Moreover, a study of the effects of Chios mastic gum on lipid and glucose metabolism of diabetic mice has shown that the resin improved lipid and glucose abnormalities.^21^

Based on the need for safe and efficacious therapy for NAFLD/NASH and the properties of Mastiha, we have investigated the effects of this resin on metabolic parameters, microbial diversity, hepatic pathology, and NAFLD activity in a biopsy‐confirmed mouse model of advanced NASH with fibrosis.

## 2 Experimental Section

### 2.1 Animals and Experimental Design

The effect of Mastiha (0.2% in the diet) was investigated in mice with diet‐induced obesity, NASH, and fibrosis (DIO‐NASH model) as described by Kristiansen et al.^22^ All animal experiments were conducted according to the international principles for care and use of laboratory animals and were covered by personal licenses for Jacob Jelsing (2013‐15‐2934‐00784 and 2015‐15‐0201‐00518) issued by the Danish committee for animal research.

A total of 34 C57BL/6J male mice (5 weeks old), obtained from JanVier (JanVier Labs, France), were included in the study. Before intervention with Mastiha, animals had ad libitum access for 41 weeks to a regular rodent diet (Altromin 1324, Brogaarden, Denmark) or a diet high in fat (40%), of these 18% trans‐fat, 40% carbohydrates (20% fructose), and 2% cholesterol (D09100301, Research Diet, USA) previously described as the AMLN diet^23^ and tap water. The AMLN diet was used for the induction of NASH in mice. Of note, following an FDA ban on trans‐fat as a food component,^24^ the NASH inducing diet has recently been changed to contain palm oil instead of trans‐fat.^25^

**Figure 1:**
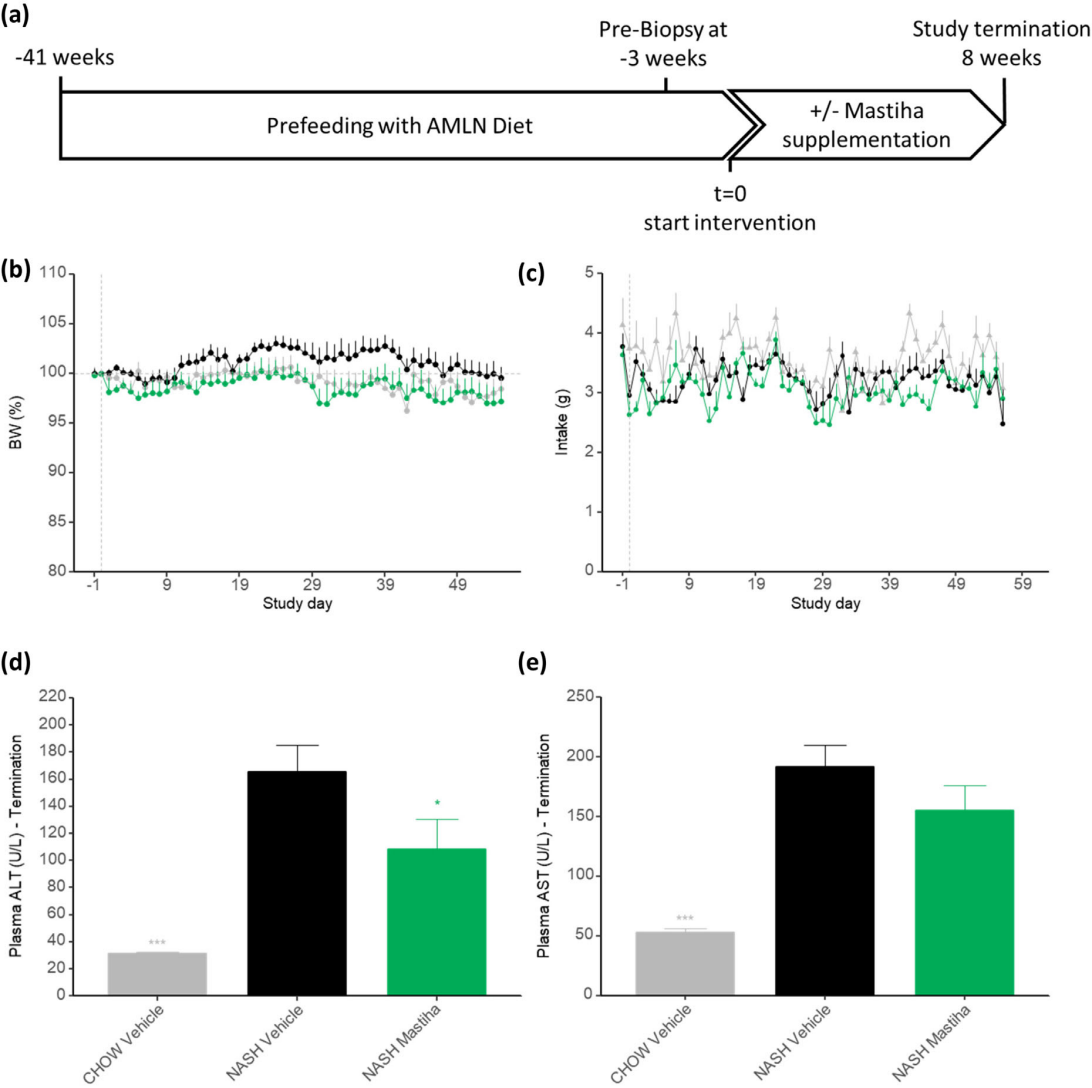
a) Study layout. b) Body weight change (% of day 0) throughout the treatment period. c) Twenty‐four hour food intake recorded daily throughout the study period. d) Plasma ALT and e) plasma AST levels at termination of the study. Values are mean of *n* = 10–11 ± SEM. ^*^ *p* < 0.05; ^***^ *p* < 0.001 compared to NASH vehicle.

The study design is illustrated in **Figure** 1a. A baseline liver biopsy was conducted 3 weeks before the intervention for histological assessment of individual fibrosis and steatosis staging, as described.^22^ A week before the intervention the animals were randomized and stratified according to liver Col1a1 quantification into three groups: Group 1, LEAN CHOW (*n* = 10); Group 2, DIO‐NASH (*n* = 12); Group 3, DIO‐NASH+MASTIHA (*n* = 12). Mice with fibrosis stage <1 and steatosis score <2 were deselected prior to randomization. The intervention lasted for a period of 8 weeks. At the end of the intervention, animals were euthanized and liver tissue and plasma were collected.

Methods used for body weight and body composition analysis, blood sampling, plasma biochemistry, endotoxin determination, and liver tissue biochemistry are detailed in Supporting Information.

### 2.2 Dosage Information

Mastiha powder was incorporated into the AMLN diet at a concentration of 0.2% (w/w). Throughout the intervention period, mice consumed about 3 g of food per day (Figure 1c). Thus, daily intake of Mastiha was ≈6 mg per animal or 160 mg kg^–1^ as the average body weight was 37.5 g at the start of intervention. This corresponds to an estimated human equivalent daily dose of 14 mg kg^–1^ 26 or 840 mg for a 60 kg person, which is similar to or below the daily doses of 1–2.8 g of Chios mastic gum that have been used in chronic clinical studies.^27^

### 2.3 Histology Assessment

Baseline liver biopsy and terminal samples were collected from the left lateral lobe (about 50–100 mg at baseline and 200 mg at the end) and fixed overnight in 4% paraformaldehyde. Liver tissue was paraffin embedded and sectioned (3 µm thickness). Sections were stained with hematoxylin and eosin and Sirius Red to assess hepatic steatosis and fibrosis, respectively, followed by analysis with Visiomorph software (Visiopharm, Denmark). Col1a1 and galectin‐3 were assessed using IHC staining. A blinded to the study pathologist performed the histological assessment and scoring. NAFLD activity score (NAS; steatosis/inflammation/ballooning degeneration) and fibrosis stage were quantified applying the criteria proposed by Kleiner et al.^28^

### 2.4 Hepatic Gene Expression Changes

Liver tissue was harvested from the left lateral lobe, stabilized overnight in RNAlater solution (Merck KGaA, Darmstadt, Germany) and stored at ‐80 °C. Total RNA isolation was performed with the miRNeasy kit following the instructions of the manufacturer (QIAGEN GmbH, Hilden, Germany). RNA was quantified with an Agilent RNA 6000 Nano kit using an Agilent 2100 Bioanalyzer (Agilent Technologies Inc, Waldbronn, Germany). Gene expression was quantified using droplet digital PCR or qRT‐PCR analysis as described in Supporting Information.

### 2.5 Fecal Microbiota DNA Extraction and Sequencing

DNA extraction from fecal pellets was performed using a NucliSENSEasyMAG platform (Biomérieux, Marcy‐l'Etoile, France) following the standard protocol. DNA was used at a concentration of 5 ng µL^–1^ in 10 mm Tris (pH 8.5) for the Illumina protocol for 16S rRNA gene Metagenomic Sequencing Library Preparation (Cod. 15044223 Rev. A). PCR primers targeting the 16S rRNA gene V3 and V4 regions were designed as in Klindworth et al.^29^ Primer sequences and details on sequencing are given in Supporting Information.

### 2.6 Bioinformatics Analysis

Quality assessment of sequencing reads was performed with the prinseq‐lite program^30^ applying the following parameters: a minimal length (min_length) of 50 nt and a quality score threshold of 30 from the 3′‐end (trim_qual_right), using a mean quality score (trim_qual_type) calculated with a sliding window of 20 nucleotides (trim_qual_window). After filtering and trimming, sequences were analyzed using the qiime2 platform.^31^ Sequence de‐noising, paired‐ends joining, and chimera depletion was performed with the DADA2 software.^32^ The taxonomic affiliations of the sequences were assigned by means of the Naive Bayesian classifier integrated in quiime2 using the SILVA_release_132 database.^33^

### 2.7 Fecal Microbiota Diversity and Composition

Then, 100%‐similarity sequence clusters were obtained and considered as Operational Taxonomic Units (OTUs) for further analyses. Diversity metrics (both phylogenetic and non‐phylogenetic) were computed within qiime2. The resulting values were compared through Kruskal‐Wallis tests. The variation in microbiota composition among treatments was visualized by means of principal coordinate analysis (PCoA) using phylogenetic and nonphylogenetic measures including the Jaccard, Bray–Curtis, and UniFrac distances.

### 2.8 Statistical Analysis

Data were presented as mean ± SEM. Statistical significance was evaluated using Dunnett's test one‐factor linear model for body composition, blood, and liver biochemistry. *T*‐test was used for the comparison of differences of gene expression between the groups. *p* < 0.05 was set as the statistical significance level. A nonparametric, two‐sided Wilcoxon's test was used for the comparison of posttreatment NAFLD activity scores.

The potential associations between gut microbiota composition and NAFLD‐related parameters were analyzed with the gneiss software within the qiime2 platform. Further details are described in Supporting Information.

## 3 Results

The effect of Chios Mastic Gum, administered orally as part of the diet (0.2%), was investigated in a chronic study in mice with diet‐induced obesity and advanced NASH with fibrosis induced by long‐term feeding with a diet rich in fat, fructose and cholesterol.^22^, ^23^ Mice were randomized to receive diet with or without Mastiha based on body weight and extent of hepatic fibrosis determined histologically in a liver biopsy taken before onset of treatment. Treatment duration was 8 weeks during which animals remained on the NASH‐inducing diet.

### 3.1 Body Composition and Food Intake

The body weight development over the 8 week treatment period was similar in mice on the NASH‐inducing diet with or without Chios mastic gum (Figure 1b). Mastiha had no significant influence on total food intake, indicating that it was well tolerated (Figure 1c). There was no significant difference in body composition between mice with or without exposure to Chios mastic gum, though mice on Mastiha showed a trend to lower relative fat mass (Figure S1, Supporting Information).

### 3.2 Biochemical Profile

Treatment with Mastiha led to a significant reduction in plasma ALT activity (Figure 1d) and a nonsignificant trend toward lower plasma AST activity (Figure 1e). While blood glucose levels were similar between the different groups, DIO‐NASH mice had significantly higher plasma insulin levels indicating that they were insulin resistant though not yet diabetic. Intake of Mastiha had no influence on glucose or insulin levels (Figure S2, Supporting Information). No significant changes were detected in plasma adiponectin between the groups (Figure S3, Supporting Information).

### 3.3 Liver Tissue Biochemistry and Histopathology

**Figure 2:**
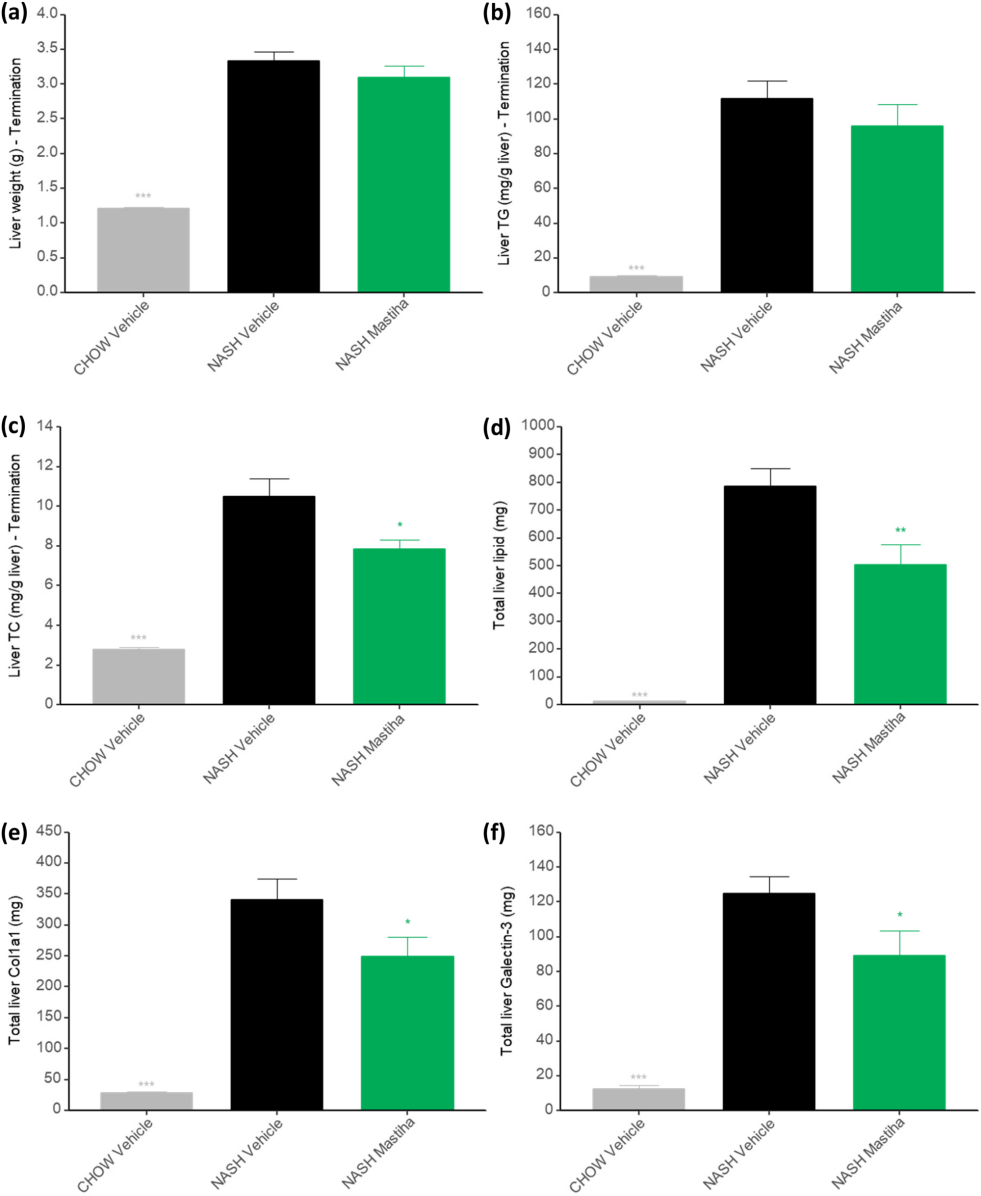
a) Liver weight, b) liver triglycerides, and c) liver total cholesterol at study termination. d) Total liver lipid content, e) hepatic col1a1, and f) hepatic galectin‐3 quantified by morphometry. *n* = 10–11 ± SEM. ^*^ *p *< 0.05; ^**^ *p* < 0.01; ^***^ *p* < 0.001 compared to NASH vehicle.

DIO‐NASH mice had enlarged livers compared to lean chow controls. Likewise, hepatic triglyceride and cholesterol levels were significantly higher. In mice exposed to Chios mastic gum, liver weights were not different from NASH control mice (**Figure** 2a), and there was a nonsignificant trend to lower hepatic triglycerides upon Mastiha treatment (Figure 2b). Notably, total hepatic cholesterol and total liver lipid were significantly reduced in Mastiha‐treated DIO‐NASH mice compared to DIO‐NASH control mice (Figure 2c,d). Additionally, hepatic Col1a1 and galectin‐3 levels were significantly reduced upon intake of Chios mastic gum, indicating a beneficial effect of treatment on liver fibrosis (Figure 2e,f and Figure S4a,b, Supporting Information).

**Figure 3:**
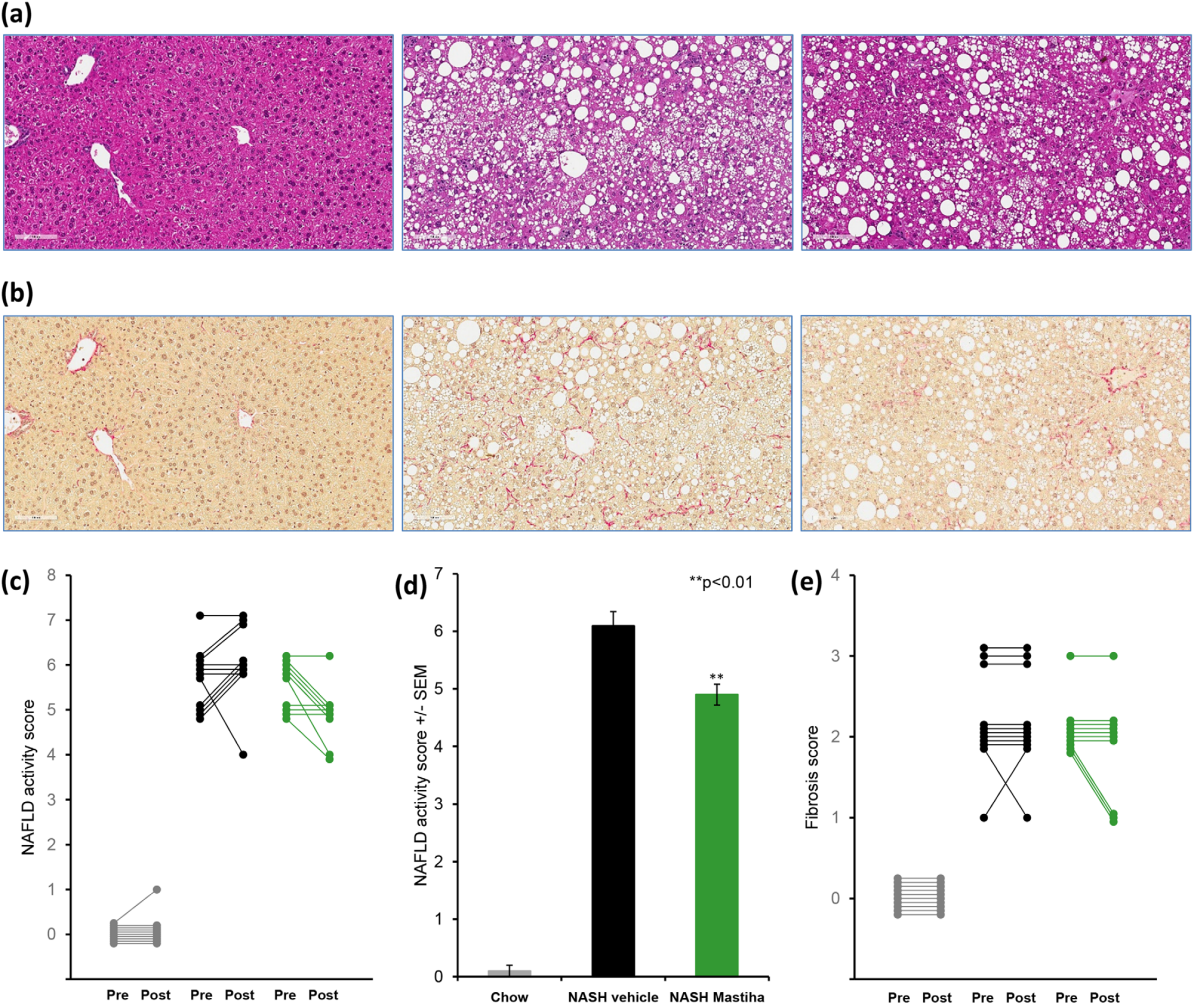
a) Representative images of liver morphology at termination of the study. H&E staining (20×, scale bar = 100 µm), b) Picrosirius Red staining (20×, scale bar = 100 µm). c) Change in NAFLD activity score (pre‐ versus posttreatment) for individual animals in the different treatment groups: Lean control mice (grey), mice on NASH diet (black), and mice on NASH diet supplemented with 0.2% Chios mastic gum (green), d) NAFLD activity score for the different treatment groups at study termination. e) Change in fibrosis score. *N* = 10–11 per group, ^**^ *p* < 0.01 versus NASH vehicle group (nonparametric two‐sided Wilcoxon's test).

Mastiha intake was associated with a significant improvement in the histological NAFLD activity score (NAS) relative to DIO‐NASH mice not exposed to Chios mastic gum (**Figure** 3). However, in DIO‐NASH controls, 54% (6/11) mice had a higher NAS after the intervention period and only 9% (1/11) showed an improvement, 60% (6/10) of Mastiha‐treated mice showed an improved NAS and no worsening of NASH was observed in the Mastiha group (Figure 3c). Following intervention, NAS was 6.1 ± 0.25 in the vehicle group compared to 4.9 ± 0.18 in the Mastiha group (*p* = 0.0019, Figure 3d). There was an improvement in individual mice relative to control NASH mice in all three components of the NAFLD activity score upon Mastiha intake (Figure S5, Supporting Information).

Fibrosis score improved in three of ten mice on Mastiha. In the NASH control group, improvement in fibrosis score was seen in one of 11 animals and worsening was observed in one of 11 mice (Figure 3e).

### 3.4 Hepatic Gene Expression

The expression of fibrosis (ACTA‐2, Col1a1, Col4a1) and inflammation (MCP‐1, TGFß‐1, TNF‐α) marker genes was significantly higher in NASH mice compared to lean chow control animals (Figure S6, Supporting Information). In line with the trend to reduced fibrosis, expression of Col1a1 and Col4a1 was reduced upon Mastiha intake, but the difference to NASH control mice was not statistically significant.

### 3.5 Gut Microbiota Diversity and Composition

**Figure 4:**
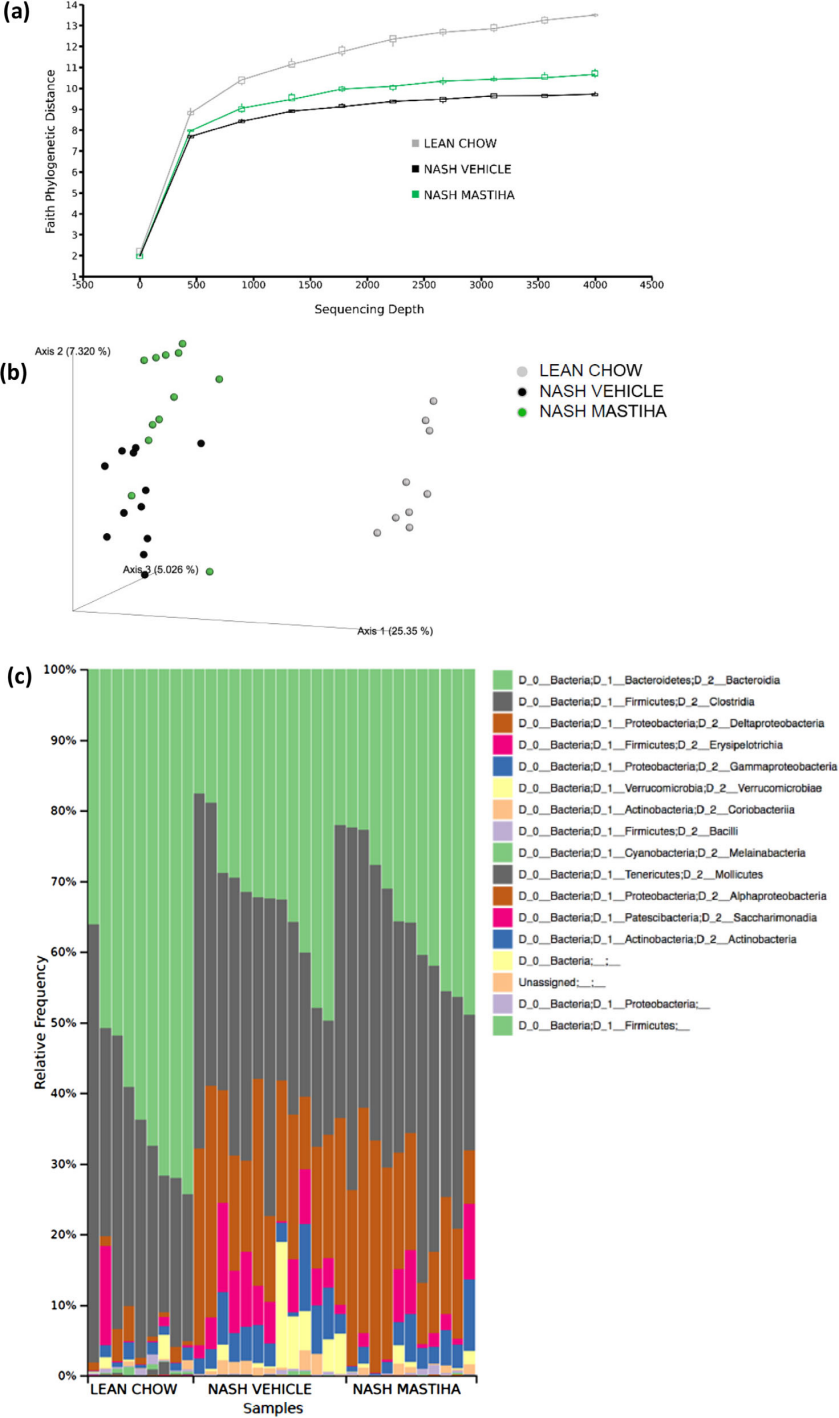
a) Faith's phylogenetic diversity (PD) index of the gut microbiota at different sequence coverages. Microbial diversity is partially recovered under Mastiha‐supplemented diet in NASH mice. b) Differences in taxonomic composition of the gut microbiota by study group: PCoA‐based on the Jaccard distance. c) Barplot of taxonomic composition at bacterial class level, showing the decrease in *Bacteroidia* and increase in *Deltaproteobacteria* abundance in NASH‐ and Mastiha‐treated mice.

**Figure** 4a presents rarefaction curves plotting Faith's phylogenetic diversity (PD) index for the microbiota of the analyzed samples at different sequence coverages, separated by study group. Microbial diversity was significantly higher in lean chow control animals in comparison to either NASH mice or Mastiha treated mice (*p* = 0.0002 in each case). However, Mastiha promoted a partial but significant recovery of diversity (*p* = 0.0496). Analyses based on other diversity measures, such as number of OTUs or Shannon's diversity index, produced similar results. Overall, the difference in microbial diversity between vehicle and Mastiha treated mice was comparably small. Changes in microbial diversity were not reflected in differences in plasma endotoxin content that was found to be similar between the three groups (Figure S7, Supporting Information).

**Figure 5:**
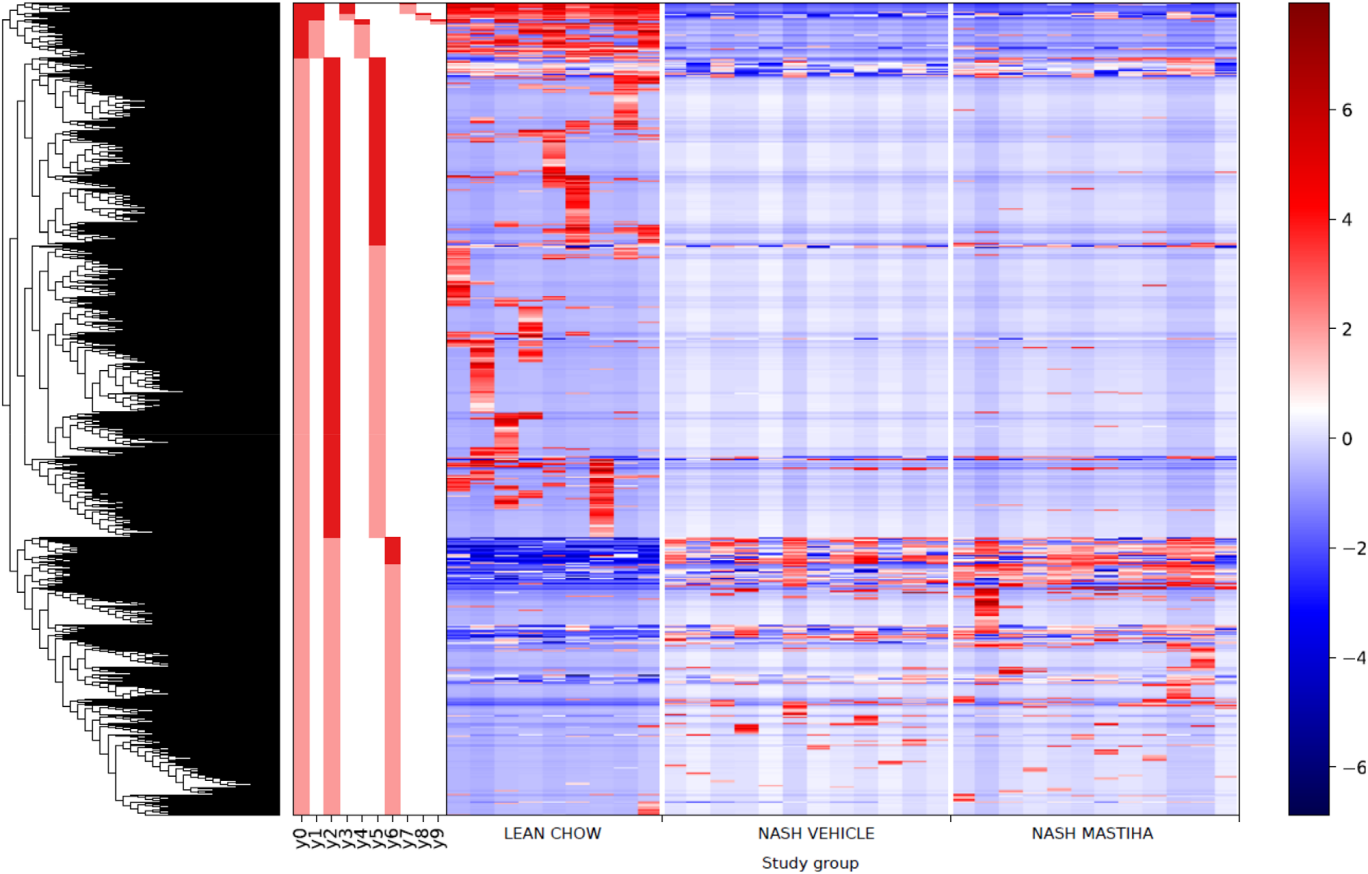
Heatmap of microbial taxa log‐scaled abundances by study group (sample mean centered around zero) and dendrogram of clustering by taxon co‐occurrence. Abundance balances were calculated as log‐abundance ratios between the groups of taxa separated by each dendrogram partition. The deepest partitions in the clustering dendrogram are indicated (*y*0 to *y*9).

Regarding the taxonomic composition of the gut microbiota, the PCoA in Figure 4b presents the variation among study groups based on Jaccard distance. Similarly to the diversity, the taxonomic composition of the gut microbiota differed most between the lean chow control animals and the other two groups, but NASH mice and Mastiha‐treated mice also tended to separate from each other along the PCo2 axis. PCoAs based on other distance measures, including the Bray–Curtis, UniFrac, and weighted UniFrac distances, showed a similar distribution of samples. When exploring the bacterial classes that predominate in the microbiota of the different study groups, we saw that *Bacteroidia* decreased in NASH‐ and Mastiha‐treated mice, whereas *Deltaproteobacteria* became more abundant (Figure 4c). The heat map in **Figure** 5 represents the abundances of the different microbial taxa present across the samples clustered according to their pattern of co‐occurrence, highlighting again the marked differences in gut microbiota composition between control animals and the other two groups, and the smaller differences between NASH‐ and Mastiha‐treated mice. In addition, the deepest partitions in the clustering dendrogram are indicated (*y*0 to *y*9). Abundance balances between the groups of taxa separated by these partitions, and all other partitions in the dendrogram, were calculated as log abundance ratios.

### 3.6 Gut Microbiota Associations with NAFLD‐Related Parameters and Gene Expression

Next, different linear regression models were applied to fit the matrix of abundance balances. We first used linear regression to evaluate the associations of this matrix with 1) study group, 2) individual NAFLD‐related biochemical and histological parameters, and 3) expression of individual genes. Table S1, Supporting Information, presents the values of *R* ^2^ for the different individual regressions. Study group was the best predictor of taxon composition, explaining 32% of the variance across samples, followed by steatosis score (28%), NAFLD activity score (26%), liver lipid content (26%), and liver triglycerides (26%). Clearly, the composition of the gut microbiota is associated with the lipid content of the liver. Regarding specific genes, Gpnmb‐Mm01328587, a type I transmembrane glycoprotein that regulates cell growth and differentiation, was most strongly linked to microbial variance (24%). Following this gene, Lpl‐Mm01345523 and Ccl2‐Mm00441242 explained 17% and 16% of the variance, and all other individual genes explain <15%.

In order to evaluate the combined explanatory capacity of the different covariates, we explored the fit of several linear regression models to the matrix of taxon abundance balances. The best model included eight predictor variables, most of which are related to liver histology (steatosis score, lobular inflammation, % Col1a1, % galectin‐3, fibrosis stage), in addition to liver tissue triglycerides (TG), plasma cholesterol (TC), and expression of the Gpnmb‐Mm01328587 gene. This model explained 45% of the variance across samples without overfitting the data. The covariates that impact the model the most were steatosis score and Gpnmb‐Mm01328587_m1 expression, each explaining 4% of the total variance as estimated by a leave‐one‐variable‐out approach.

The regression analyses defined a series of *p*‐values for the correlation coefficients of each analyzed predictor variable with specific partitions of the tree depicted in Figure 5. The taxonomic composition of the numerator and denominator in a partition that associates significantly with a given variable gives an indication of which specific bacterial taxa are likely changing in abundance in response to that variable. The *y0* partition associated significantly with study group (fdr‐corrected *p* = 5.88 × 10^–11^) and with several biochemical, histological, and gene expression variables in simple regressions, including steatosis score (fdr‐corrected *p* = 2.01 × 10^–12^), NAFLD activity score (fdr‐corrected *p* = 3.90 × 10^–04^), %liver lipid (fdr‐corrected *p* = 1.24 × 10^–19^), liver triglycerides (fdr‐corrected *p* = 9.52 × 10^–19^), plasma ALT (fdr‐corrected *p* = 7.72 × 10^–13^), and Ccl2_Mm00441242 expression (fdr‐corrected *p* = 1.06 × 10^–04^). The ratio was significantly lower in the control group, as the taxa in the *y0_numerator_* were on average less abundant than the taxa in the *y0_denominator_*, whereas the reverse was true for most NASH mice and Mastiha‐treated mice (Figure S8a, Supporting Information). Moreover, Mastiha treatment had little effect on the ratio of these taxa. Figure S8b, Supporting Information, shows the *y0_numerator_* and *y0_denominator_* taxa that varied the most between control mice, on the left, and NASH‐ and Mastiha‐treated mice, on the right. In NASH‐ and Mastiha‐treated mice, an uncultured *Deltaproteobacteria* of the *Desulfovibrionaceae* family was highly over‐represented in comparison to control mice, whereas several *Bacteroidiae* of the family *Muribaculaceae* were under‐represented, in accordance with the class level bar plots in Figure 4c.

## 4 Discussion

The prevalence of NAFLD/NASH is increasing worldwide in parallel with obesity, type 2 diabetes mellitus and metabolic syndrome. It is estimated that NAFLD/NASH will be the leading cause of cirrhosis and hepatocellular carcinoma in the next 5 years.^34^ Due to the absence of targeted pharmacological therapy, there is an increasing trend in the evaluation of the effectiveness of natural products in combination with lifestyle dietary changes.

Mastiha is a natural food supplement rich in phytochemicals with preclinical and clinical data proving its antioxidant and anti‐inflammatory properties. The supplement has been studied in various pathologies, such as Crohn's disease,^19^, ^20^ *Helicobacter pylori* infection,^35^ and hypercholesterolaemia.^18^ Another study in streptozocin‐induced diabetic mice evaluating the impact of Mastiha's administration on lipid and glucose metabolism showed that this resin improved lipid and glucose abnormalities and partially reversed hepatic damage.^21^

To our knowledge, this is the first study investigating the effect of Mastiha in obese, insulin‐resistant mice with NASH and fibrosis. Etiology of NASH in this model is very similar to what is seen in humans, where a large proportion of individuals with NASH is obese and insulin‐resistant or even diabetic.^2^ Importantly, our study was designed as an intervention study, with treatment commencing at a point where advanced steatohepatitis with fibrosis was already present. The daily dose of Chios mastic gum corresponded to a human equivalent dose similar to the doses used in chronic clinical studies. Moreover, the mice remained on the NASH‐inducing diet throughout the 8 week treatment period. Therefore, it is remarkable that supplementation with Chios mastic gum led to a significant reduction in plasma ALT activity, hepatic steatosis, and the histological NAFLD activity score. Of note, the marked improvement in liver pathology occurred in the absence of weight loss, suggesting a direct effect on the liver rather than an indirect effect of reduced adiposity.

This is also the first study investigating the effect of Mastiha on gut microbiota composition. Supplementation with Chios mastic gum led to a significant recovery of gut microbiota diversity in obese, insulin‐resistant mice with NASH and fibrosis, although not sufficient to recover the levels of diversity observed in lean control mice. Furthermore, Mastiha supplementation resulted in small changes in the identity and abundance of the specific microbial taxa but could not reverse the major differences between NASH mice and lean control animals. In particular, the development of NASH was accompanied by a strong reduction of the *Muribaculaceae* family (previously called the S24‐7), which commonly dominates the mouse fecal microbiota, and the expansion of a single bacterium of the *Desulfovibrionaceae* family. Notably, *Muribaculaceae* degrade dietary and host glycans and other polysaccharides to produce SCFAs such as succinate, acetate, and propionate. SCFAs are known to act as intermediaries between the gut microbiota and host physiology as they have important anti‐inflammatory properties as well as effects on glucose and lipid homeostasis.^36^ The *Desulfovibrionaceae*, on the other hand, have been shown to significantly increase in obese humans compared to lean individuals^37^ and blooms of this family have been demonstrated in mouse models of diet‐induced obesity and diabetes.^38^, ^39^ It is thought that the LPSs produced by these bacteria lead to the low‐grade inflammation associated to these diseases. Therefore, the decrease in *Muribaculaceae* and the concomitant increase in *Desulfovibrionaceae* may clearly have contributed to several of the phenotypes associated with NASH development. The fact that microbiota composition, and in particular the ratio of *Desulfovibrionaceae* to *Muribaculaceae* reflected in the *y0* partition, correlated significantly with parameters such as steatosis score, NAFLD activity score, %liver lipid, liver triglycerides, plasma ALT, and Ccl2_Mm00441242 expression reinforces the notion that alterations in the abundance of these bacteria have an impact on histological, biochemical, and gene expression parameters of relevance in NASH. Of note, changes in microbial composition upon treatment with Mastiha have been rather small, and associations between microbiota changes and NASH improvement do not necessarily indicate a cause‐and‐effect relationship. Further studies, e.g., using other interventions improving NASH, will be required to demonstrate causality.

The model used in our study has several strengths. First, NASH is induced by chronic overnutrition using a diet rich in saturated fats and simple carbohydrates, especially fructose. This is similar to what drives development of NAFLD and NASH in people. Second, Mastiha supplementation started when NASH and fibrosis were already manifested. Third, biopsies were taken before and after intervention with Chios mastic gum, allowing for monitoring the change in liver histology on the level of individual animals. Fourth, the histological scoring system used in our study was the same that is used clinically to diagnose and grade NASH and fibrosis. Limitations of the model are its dependence on high dietary cholesterol (2%) and the lack of advanced hepatocyte ballooning injury. Therefore, our results may not be directly extrapolated to humans. Since previous research has shown that Mastiha's main bioactive compounds, namely terpenes, are bioavailable in humans and exhibit antioxidant effects,^40^ further studies on NAFLD patients are of particular interest.

In conclusion, this is the first study, according to our knowledge, evaluating the effects of Mastiha administration on an animal model of NAFLD/NASH showing promising results on liver steatosis and NAFLD activity. Further studies unravelling the mechanisms of action are necessary.

## Conflict of Interest

A.K., C.K., M.S., and U.S. are employees of Sanofi. A.N.M. and M.V.O. are employees of Gubra.

## Supporting information

Supporting Information
