# Supplementary material for: Mastiha (Pistacia lentiscus) Improves Gut Microbiota Diversity, Hepatic Steatosis, and Disease Activity in a Biopsy‐Confirmed Mouse Model of Advanced Non‐Alcoholic Steatohepatitis and Fibrosis
Source: Mol Nutr Food Res. 2019 Oct 23;63(24):1900927. doi: 10.1002/mnfr.201900927 (PMC7003480; doi:10.1002/mnfr.201900927)
Supplement: Supplementary file 1 — Supporting Information [file MNFR-63-1900927-s001.docx]

**SUPPORTING INFOMRATION to**

**Mastiha (*Pistacia lentiscus*) improves gut microbiota diversity, hepatic steatosis and disease activity in a biopsy-confirmed mouse model of advanced non-alcoholic steatohepatitis and fibrosis.**

Aimo Kannt, Efstathia Papada, Claire Kammermeier, Giuseppe D’Auria, Nuria Jiménez-Hernández, Martin Stephan, Uwe Schwahn, Andreas Nygaard Madsen, Mette Viberg Ostergaard, George Dedoussis, M Pilar Francino on behalf of the MAST4HEALTH consortium

**SUPPORTING METHODS**

**Body weight and body composition analysis**

Body weight was monitored once daily during the intervention period. Whole-body fat mass was analyzed week 7 of the intervention period by non-invasive EchoMRI scanning using EchoMRI-900 (EchoMRI, United States). During the scanning procedure the mice were placed in a restrainer for 60-90s.

**Blood sampling and plasma preparation**

Intermediate plasma samples were obtained from the tail vein of conscious animals at week +6 for 4h fasting glucose and 4h fasting plasma insulin quantification. At the end of the study, during anesthesia with isoflurane, the abdominal cavity was opened and cardiac blood was drawn with a regular syringe into EDTA tubes or with a coated (heparin/EDTA) vacutainer. Blood was placed at 4°C until it was centrifuged at 2000 g for 10 minutes. The plasma supernatants were transferred to new tubes and immediately frozen on dry ice and stored at -80°C.

**Plasma Biochemistry analysis**

Mouse 4h fasting insulin was measured using the MSD platform (Meso Scale Diagnostics) according to the manufacturer’s instructions and 4h fasting glucose was measured using a Biosen c-line glucose meter (EKF-diagnostics, Germany). Mouse plasma adiponectin was measured at the end of intervention using the ELISA technique (R&D Systems) according to the manufacturer’s instructions. Triglycerides (TG), Total cholesterol (TC), Alanine transaminase (ALT), Aspartate transaminase (AST) were measured at the end of intervention using commercial kits (Roche Diagnostics, Germany) on the Cobas™ C-501 autoanalyzer according to the manufacturer’s instructions.

**Plasma endotoxin content determination**

Plasma endotoxins were quantified using a competitive EIA (Gram Negative Endotoxin ELISA Kit, LifeSpan Biosciences Inc., USA) according to the manufacturer’s instructions.

**Liver tissue biochemistry**

The triglyceride (TG) content in liver was determined using the Triglyceride reagent (Cat. no. 22-045-795, Roche Diagnostics, Germany) and the cholesterol (TC) content in liver was determined using the Cholesterol reagent (Cat. no. 22-045-780, Roche Diagnostics, Germany) on the Cobas™ C-501 autoanalyzer. Homogenized liver tissue was heated to 80-100°C twice, centrifuged in a microcentrifuge and the triglyceride and cholesterol content was measured in the supernatant.

**Gene expression analysis**

The expression of the genes encoding Collagen Type 1 Alpha 1 (Col1a1), Collagen Type 4 Alpha 1 (Col4a1), Transforming Growth Factor beta-1 (TGFß-1), Tumor Necrosis Factor alpha (TNF-α), C-C Motif Chemokine Ligand 2 (CCl2, also known as Monocyte Chemoattractant Protein 1, MCP-1) and Actin alpha-2 (ACTA-2) was quantified applying two-step droplet digital Polymerase Chain Reaction (ddPCR). The iScript™ cDNA synthesis kit (Bio-Rad GmbH, München, Germany) was used for the cDNA generation according to the manufacturer’s instructions in a T100 thermal cycler (Bio-Rad). The ddPCR™ Supermix for probes (no dUTP, Bio-Rad) was used for the Droplet Digital™ PCR (ddPCR). After droplet generation with the Automatic Droplet Generator (Bio-Rad), thermal cycling took place followed by reading of the droplets on a QX200 Droplet Reader (Bio-Rad).

For microfluidic card qRT-PCR analysis 1µg of total RNA was used for cDNA synthesis with the Quantitect^TM^ Reverse Transcription Kit from Qiagen (Hilden, Germany) according to the manufacturer’s instructions and subsequently run on a ViiA7^TM^ Real Time PCR 384 well cycler and fluorescence plate reader using the indicated primer probe pairs from Thermo Fisher Scientific, Darmstadt, Germany.

Results were expressed as the normalized ratio of copies of the gene of interest to the copies of the housekeeping gene (herein Actin Beta, ACTB).

**Fecal microbiota DNA sequencing**

The following primer sequences targeting the 16S rRNA gene V3 and V4 regions were used:

Forward 5’TCGTCGGCAGCGTCAGATGTGTATAAGAGACAGCCTACGGGNGGCWGCA-G3’

Reverse 5’GTCTCGTGGGCTCGGAGATGTGTATAAGAGACAGGACTACHVGGGTATCTAATCC3’.

Primers contained adapter overhang sequences added to the gene-specific sequences, making them compatible with the Illumina Nextera XT Index kit (FC-131-1096). After 16S rRNA gene amplification, amplicons were multiplexed and 1 ml of amplicon pool was run on a Bioanalyzer DNA 1000 chip to verify amplicon size (~550 bp). After size verification, libraries were sequenced in an Illumina MiSeq sequencer according to manufacturer’s instructions in a 2x300 cycles paired-end run (MiSeq Reagent kit v3 MS-102-3001).

**Statistical analysis of associations between gut microbiota composition and NAFLD**

The potential associations between gut microbiota composition and NAFLD-related parameters were analyzed with the gneiss software within the qiime2 platform. Gneiss performs differential abundance analysis using balances to identify differentially abundant taxa in a compositionally coherent way. Rather than focusing on individual taxa proportions, gneiss focuses on the ratios between taxa or groups of taxa, which facilitates the identification of the actual microbes that are changing. To this aim, taxa were first clustered based on how often they co-occur with each other via Ward’s hierarchical clustering. Then, for each sample, a balance was obtained at each node of the clustering dendrogram by computing the log abundance ratio for the taxa underneath that node. Statistical procedures were then performed using the obtained balances rather than crude taxon abundances or proportions.

Multivariate response linear regression models were built to predict the matrix of abundance balances depending on the different NAFLD-related covariates. The covariates employed in the models included parameters of plasma biochemistry (ALT and AST activity, TG, TC, insulin), liver tissue biochemistry (TG, TC, hydroxyproline), liver histology (Col1a1, galectin-3, steatosis, lobular inflammation, hepatocellular ballooning, fibrosis stage, NAS) and liver gene expression.

**Table S1. R^2^ in simple regression for study group and for histological, biochemical and gene expression variables explaining >10% of the variance in abundance ratios of gut microbiota taxa**

| **Variable** | **R^2^ in simple regression** |
| --- | --- |
| Study group | 0.32 |
| Post Steatosis score | 0.28 |
| Post NAFLD activity score | 0.26 |
| % liver lipid | 0.26 |
| Liver TG (mg/g liver) | 0.26 |
| Post Lobular inflammation | 0.24 |
| Total liver lipid (mg) | 0.24 |
| Post Fibrosis stage | 0.24 |
| Liver TG (mg/liver) | 0.24 |
| Gpnmb-Mm01328587_m1 | 0.24 |
| Plasma TC (mmol/L) | 0.24 |
| Plasma ALT (U/L) | 0.23 |
| % Galectin-3 | 0.22 |
| Liver TC (mg/g liver) | 0.23 |
| Plasma AST (U/L) | 0.21 |
| % Col1a1 | 0.23 |
| Total liver Col1a1 (mg) | 0.20 |
| Total liver Galectin-3 (mg) | 0.19 |
| Liver TC (mg/liver) | 0.18 |
| Lpl-Mm01345523_m1 | 0.17 |
| Ccl2-Mm00441242_m1 | 0.16 |
| Liver HP (µg/liver) | 0.15 |
| Liver HP (µg/mg liver) | 0.14 |
| Cyp7b1-Mm00484157_m1 | 0.13 |
| Post Hepatocellular ballooning | 0.13 |
| Pcsk9-Mm01263610_m1 | 0.12 |
| Homer2-Mm01314936_m1 | 0.12 |
| Slc51b-Mm01175040_m1 | 0.11 |

**(a) (b)**

**
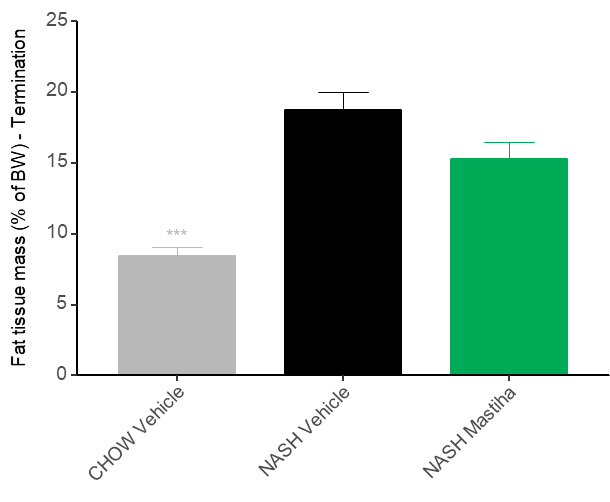

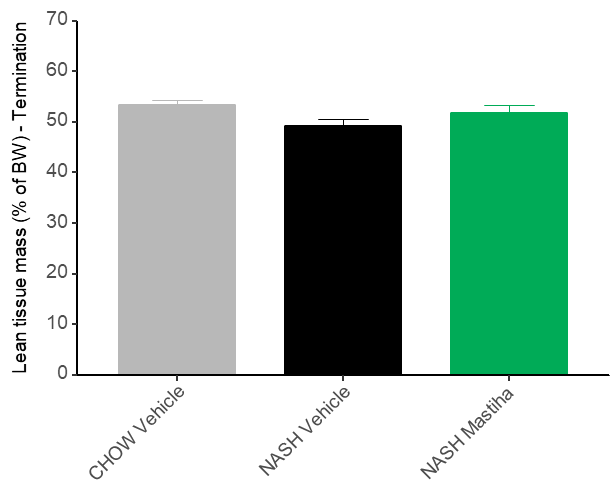
**

**Figure S1. (a)** Relative fat tissue mass and **(b)** relative lean tissue mass at study week 7 (one week before termination). Values are mean of n=10-12, ***p<0.001 compared to NASH Vehicle

**(a) (b)**

**
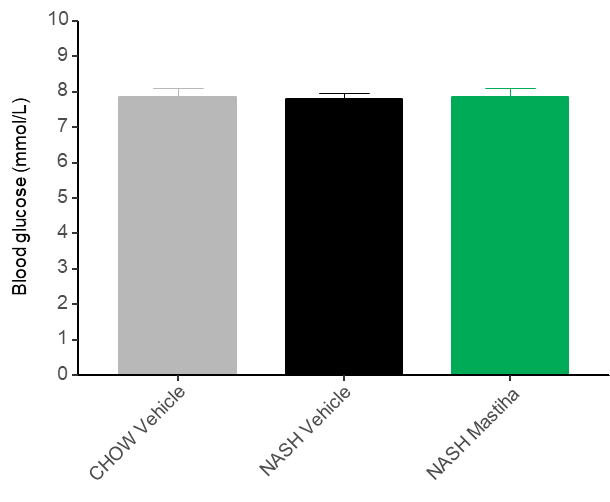

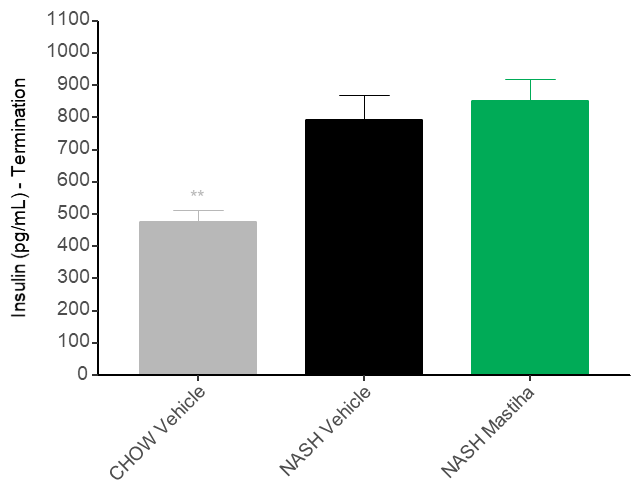
**

**Figure S2. (a)** Blood glucose and **(b)** plasma insulin (right) after 4h of fasting at study week 6. Values are mean of n=10-12 + SEM. **p<0.01 compared to NASH Vehicle

**
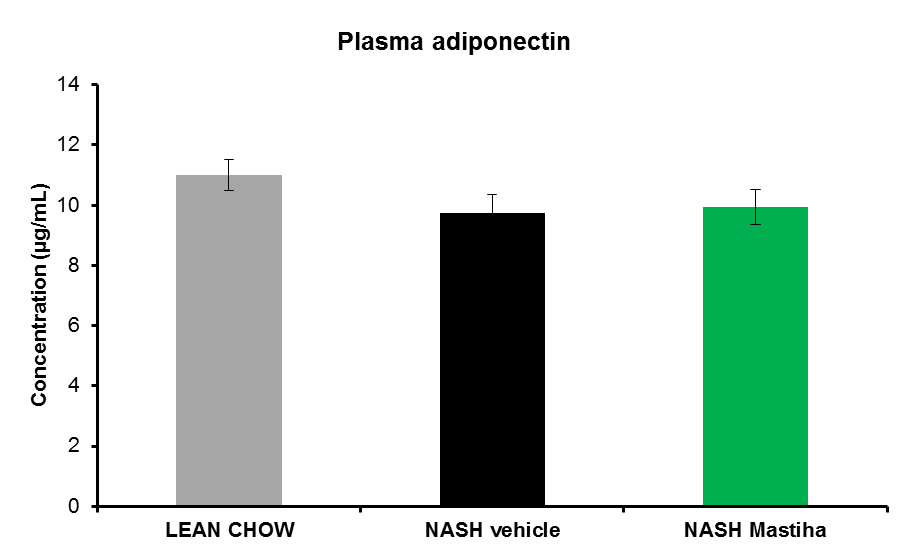
**

**Figure S3:** Plasma adiponectin levels at termination of the study.

**(a)**

**
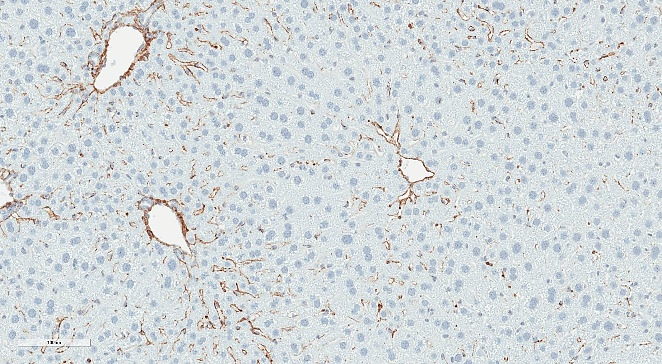

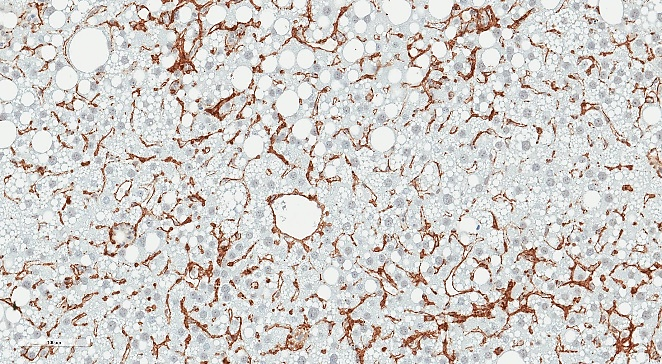

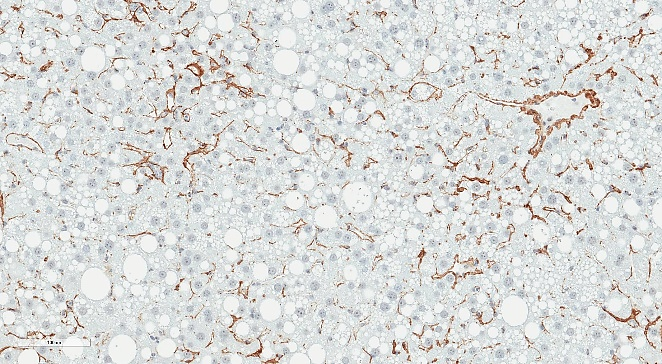
**

**(b)**

**
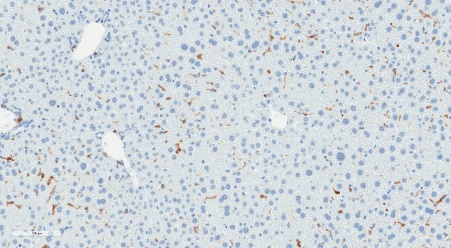

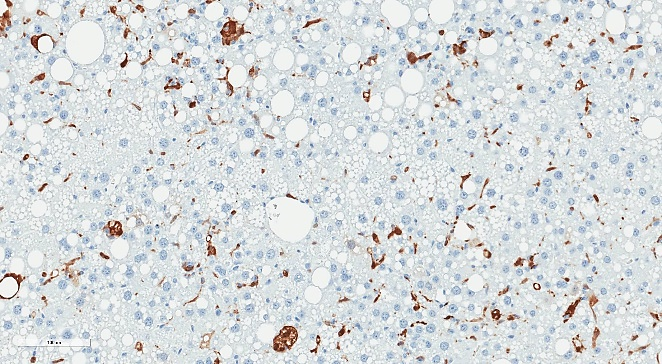

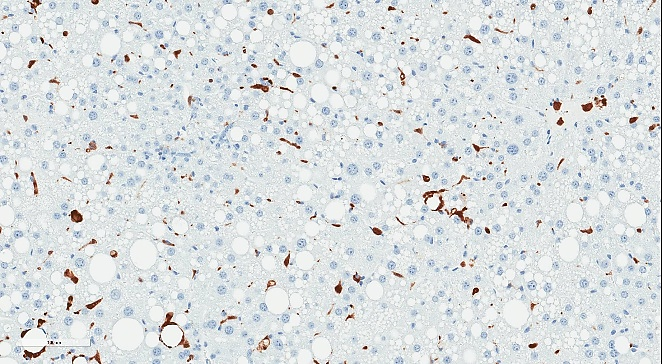
**

**Figure S4.** Representative images of liver stained with **(a)** anti-type I Collagen (Col1a1) (Southern Biotech, cat. no. 1310-01) or **(b)** with anti-Galectin-3 (Biolegend, cat. 125402) at the end of treatment period (magnification 20x, scale bar = 100 μm). ***Left:*** Chow control group. ***Middle:*** NASH vehicle group. ***Right:*** NASH Mastiha group

**(a)**

**
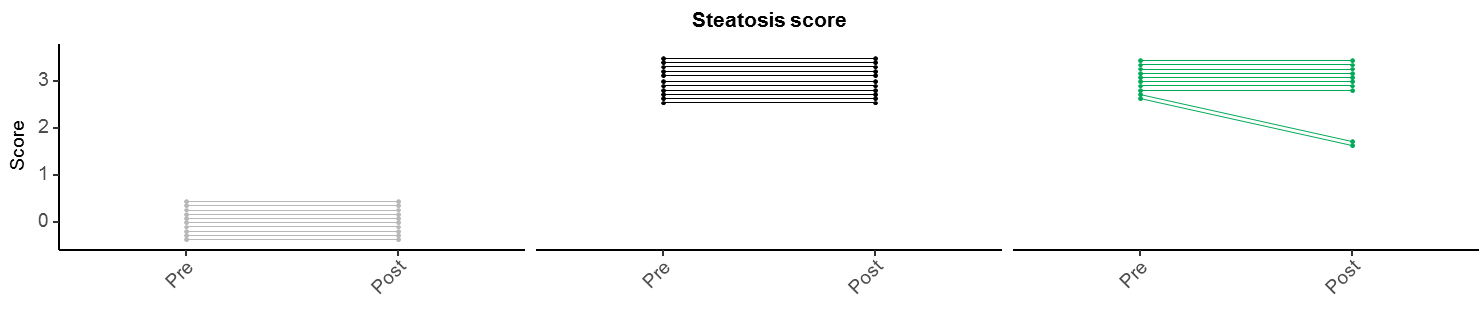
**

**(b)**

**
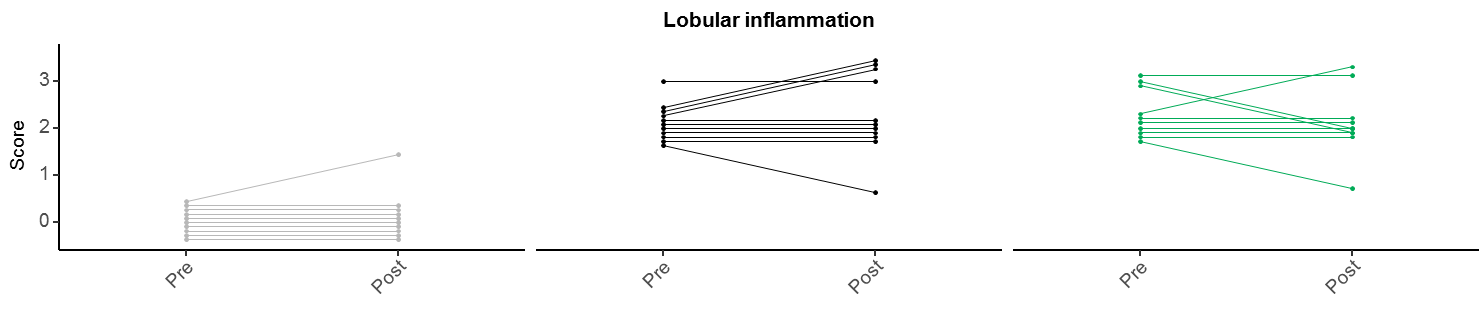
**

**(c)**

**
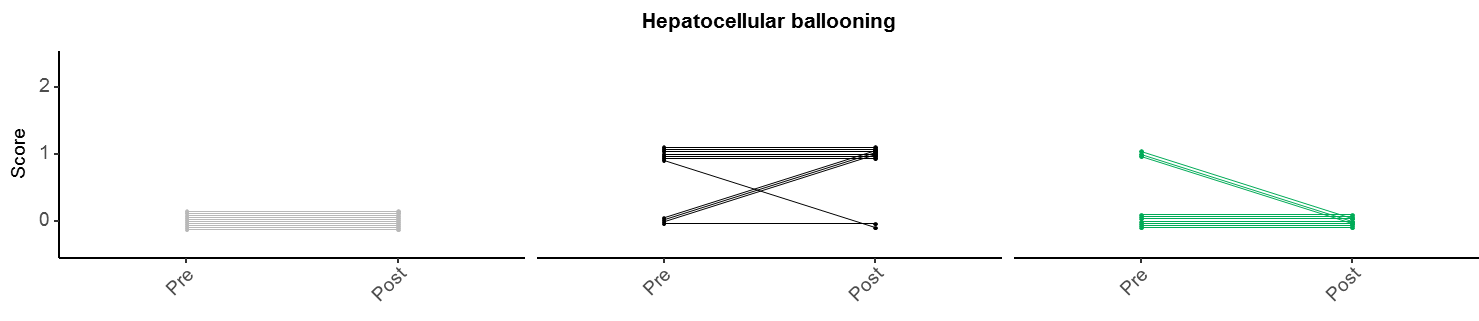
**

**Figure S5:** Changes in histological **(a)** steatosis, **(b)** lobular inflammation, and **(c)** hepatocellular ballooning scores between the pre-biopsy taken three weeks before study start (“pre”) and biopsies taken at study termination (“post”).

**
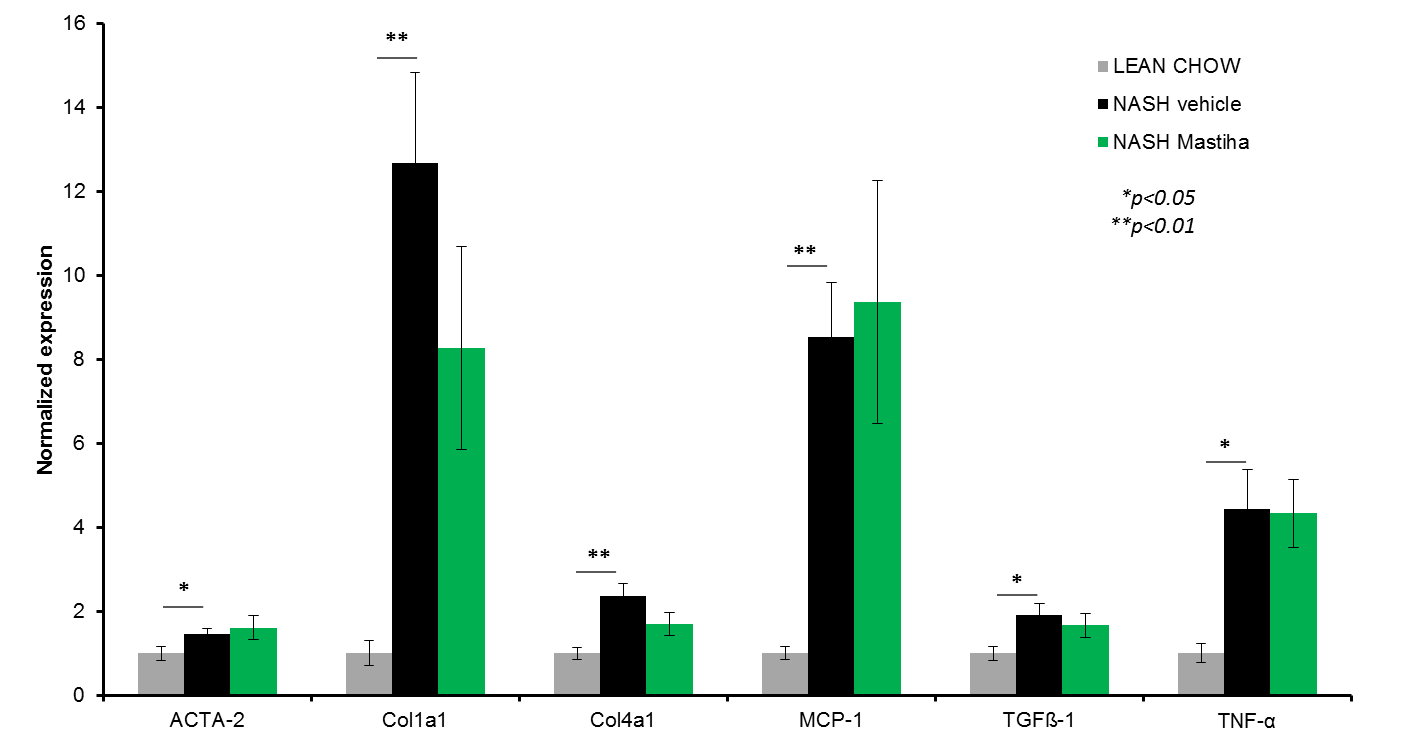
**

**Figure S6.** Expression of fibrosis and inflammation marker genes in the livers of lean controls (grey), mice on NASH diet (black) and mice on NASH diet supplemented with 0.2 % Chios mastic gum. N=10-11 per group. *p<0.05, **p<0.01 vs NASH vehicle group


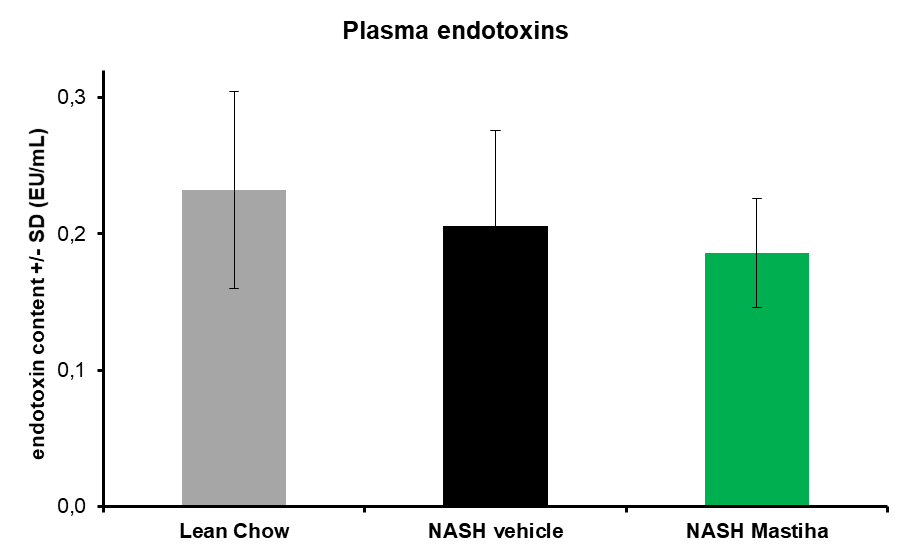


**Figure S7.** Endotoxin content in terminal plasma. Values are shown as endotoxin units per ml. Error bars denote standard deviation. N=10-11 per group.

**(a)**

**
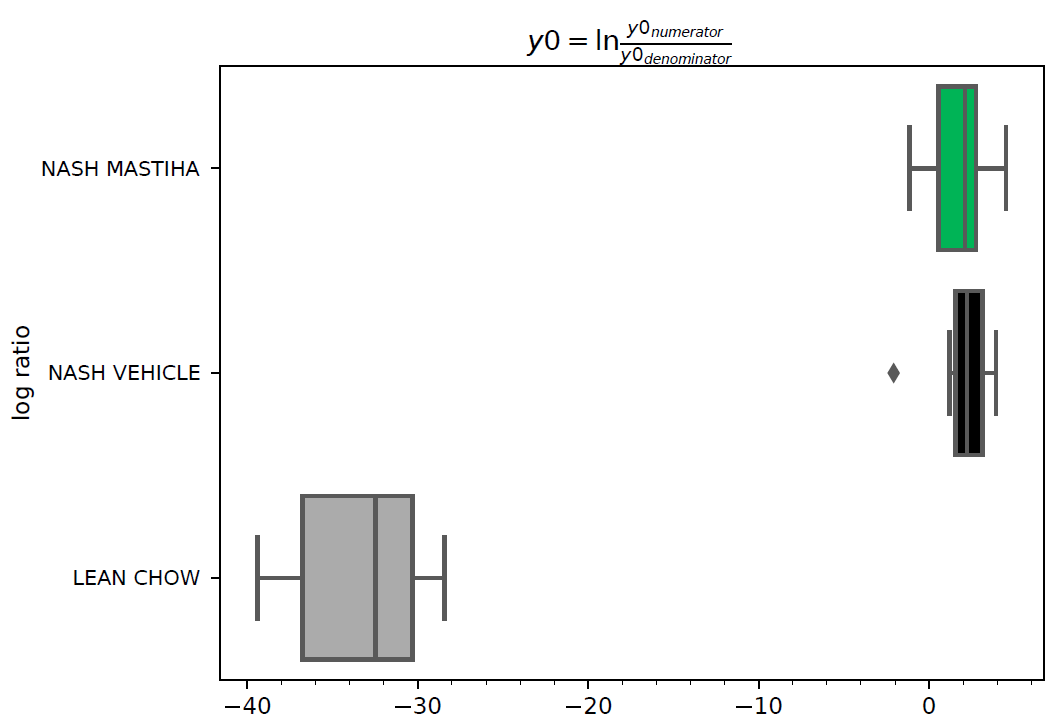
**

**(b)**

**Figure S8.** (**a**) Boxplots of log-abundance ratios by study group for the *y0* partition. (**b**) Proportion plot of the *y0_numerator_* (light blue) and *y0_denominator_* (dark blue) taxa that vary the most between control mice, on the left, and NASH and Mastiha-treated mice, on the right. The families to which each of these taxa belong are reported. Note that a single taxon belonging to the *Desulfovibrionaceae* family is highly over-represented in NASH and Mastiha-treated mice, whereas several different taxa belonging to the *Muribaculaceae* family are over-represented in control mice.
